# Reality check of using the surgical safety checklist: A qualitative study to observe application errors during snapshot audits

**DOI:** 10.1371/journal.pone.0203544

**Published:** 2018-09-06

**Authors:** Gerald Sendlhofer, David Benjamin Lumenta, Gudrun Pregartner, Karina Leitgeb, Peter Tiefenbacher, Veronika Gombotz, Christian Richter, Lars Peter Kamolz, Gernot Brunner

**Affiliations:** 1 Executive Department for Quality and Risk Management, University Hospital Graz, Graz, Austria; 2 Research Unit for Safety in Health, c/o Division of Plastic, Aesthetic and Reconstructive Surgery, Department of Surgery, Medical University of Graz, Graz, Austria; 3 Institute for Medical Informatics, Statistics and Documentation, Medical University of Graz, Graz, Austria; Nord University, NORWAY

## Abstract

**Background:**

The WHO Surgical Safety Checklist (SSC) was established to address important safety issues and to reduce the number of surgical deaths. So far, numerous reports have demonstrated sub-optimal implementation of the SSC in practice and limited improvements in patient outcomes. Therefore, the aim of this study was to audit the SSC-practice in a real-world setting in a university hospital setting.

**Methods:**

From 2015 to 2016, independent observers performed snapshot audits in operating theatres and shadowed the three phases of the SSC. Using a 4-point Likert-scale to rate the compliance on each audit day, we generated a report highlighting possible improvements and provided feedback to the operating team members.

**Results:**

Audits were performed on 36 operating days (2015: n = 19; 2016: n = 17), in which a total of 136 surgical interventions were observed. Overall, the percentage of “very good compliance” improved from 2015 to 2016: for the sign-in from 52.9% to 81.2% (p = 0.141), for the team-time-out from 33.3% to 58.8% (p = 0.181), and for the sign-out from 21.4% to 41.7% (p = 0.401). The qualitative review revealed inconsistencies when applying the SSC, of which the missing documentation of an actually performed item or the wrong timing for an item was most common.

**Conclusion:**

Snapshot audits revealed that SSC compliance has improved over the observed period, while its application revealed inconsistencies during the three phases of the SSC. Snapshot audits proved to be a valuable tool in the qualitative analysis of SSC compliance and gave more insight than a mere completeness check of ticks in SSC documents.

## Introduction

Medical errors were the purported “third leading cause of death” in hospitals in the US in 2016 [1]. Among numerous initiatives aiming to improve clinical processes, the WHO Surgical Safety Checklist (SSC) became one of the most commonly recommended tools for risk management worldwide [2–4]. Only its adequate application seemed to achieve the envisaged goal of reducing morbidity and mortality following surgical procedures [4–8]. However, SSCs were also reported to be used incompletely and merely as a tick-off list for monitoring purposes, hindering the usual processes in the operating theatre (OR), and notably failing to demonstrate clinical improvements [9–15].

A recent study showed that individuals bearing management responsibility had more positive attitudes towards the SSC than individuals without it [16]. Barriers to proper SSC application also included insufficient instruction prior its implementation, perceived redundancy of collected data and poor team culture [17]. Our hospital performs approximately 47,000 surgical procedures in 44 operating theatres each year and a previous in-house survey among healthcare professionals revealed a high level of perceived usefulness of the SSC, while compliance rates, assessed by paper-based audits, remained low [10,11]. These audits were performed by independent observers on two defined days by collection and review of paper-based hospital-wide SSCs. According to these audits, SSCs were used in 93.1% of all operations; however, just 57.2% were completed [11]. The approach of collecting quantitative data through audits is one possible approach to check SSC compliance, but how is the SSC used in reality?

In a previous publication snaphot audits were introduced as a method to gain further insight into the application of patient-safety relevant topics in daily clinical practice (eg. on wards or the OR) [11,18]. The aim of the present study was to apply the methodology of snapshot audits with incorporated feedback sessions for evaluating surgical teams in two successive years. Furthermore, it was our aim to evaluate the general suitability of snapshot audits in the OR-setting.

## Materials and methods

Following ethical board approval (Medical University of Graz, vote# 29–328 ex 16/17), we planned snapshot audits in 11 department (n = 44 ORs) in two consecutive years (2015 and 2016).

All snapshot audits were approved by the upper management. Since snapshot audits were introduced as a routine quality assurance programme, no separate informed consent was needed by observed healthcare professionals in the respective OR. Snapshot audits were announced in due time to department leaders and they informed their colleagues about the Executive Department for Quality and Risk Management performing the snapshot audit.

### Snapshot audit

In general, if guidelines are properly applied as intended can be either evaluated using a quantitative approach (paper-based audits) or through a qualitative approach such as using the method of direct observations (snapshot audits) [10, 11, 18]. In order to perform snapshot audits a checklist for observers is needed. Therefore, a checklist according to the implementation manual of the WHO SSC “Safe Surgery Saves Lives” was developed (Table 1) [19]. In 2015 and 2016, the observation of an operation started by two independent and trained observers when the patient entered the transfer area of the OR, and ended after the patient left the OR. All surgical departments/divisions were included for snapshot audits. The following phases (Table 1) were assessed:

- Before induction of anaesthesia (Sign-in (SI))
- Before skin incision (Team-time-out (TTO))
- Before patient leaves operating room (Sign-out (SO)).

**Table 1:** Checklist and reminders for the observers.

**Checklist**	
**Likert-Scale**	1 = very good compliance; 2 = good compliance; 3 = rather good compliance; 4 = non-compliant; NA = not observed
**Sign-in (before induction of anaesthesia)**	
	SI performed in the transfer area?
Reminder: • Were questions asked and checklist items ticked off immediately afterwards? • Comments for the audit team (observed errors)	
**Team-time-out (before skin incision)**	
	TTO performed before skin incision?
Reminder: • Was there an audible announcement of the TTO by an OR team member? • Were routine activities stopped by the OR team? • Were checklist items ticked off immediately afterwards? • Were answers audibly verified with immediate written documentation upon response? • Did the team paid attention and focussed on the TTO? • Was the end of the TTO audible announcement? • Comments for the audit team (observed errors)	
**Sign-out (before patient leaves the operating room)**	
	SO performed before skin suture?
Reminder: • Was there an audible announcement of the SO by an OR team member? • Were routine activities stopped by the OR team? • Were checklist items ticked off immediately afterwards? • Did the team paid attention and focussed on the SO? • Was the end of the SO audible announcement? • Comments for the audit team (observed errors)	

The three checkpoint items were rated on a 4-point-Likert-scale indicating “very good compliance”, “good compliance”, “rather good compliance” or “non-compliance”. These ratings referred to >75%, 74–50%, 49–25%, and <25% completion rates on the audit day, respectively. A completion rate of >75% indicated that SSCs were performed as intended on the observed day. In such cases the SSC was used at the right time and as intended according to the WHO implementation manual. 74–50% indicated that in some of the observed operations the SSC was not performed as intended. Such cases could have been missing checks during any of the three phases of the SSC. 49–25% were rated in cases when major parts of the SSC were not done properly. Non-compliant was rated if the SI, TTO or SO were not done at all.

Each surgical intervention was observed by two independent observers from the Executive Department for Quality and Risk Management. A checklist including reminders was used and comments after each of the three phases of the SSC were noted (Table 1). For each of the three SSC phases, observers recored how well each checklist item of the in-house hardcopy SSC was performed by the OR team. In general, the WHO-SSC divides the operation in three phases, each corresponding to a specific time period in the normal flow of a procedure, namely the above mentioned SI, TTO and SO [19]. Within each of the three phases, certain questions need to be checked. The in-house SSC was modified to fit local practice as encouraged by the WHO [10, 19]. The SI has to be performed by the scrub nurse, anesthesia nurse and the anesthesiologist. The TTO and the SO has to be initiated by the surgeon. The circulating nurse as the designated checklist coordinator has to guide the team throughout all questions and to tick the corresponding checkboxes. The checklist coordinator only ticks the checkbox if an answer was given to the corresponding question [10, 11]. Both observers were trained to review the SSC application in practice according to the requirements as stated by the WHO [19].

**Fig 1:**
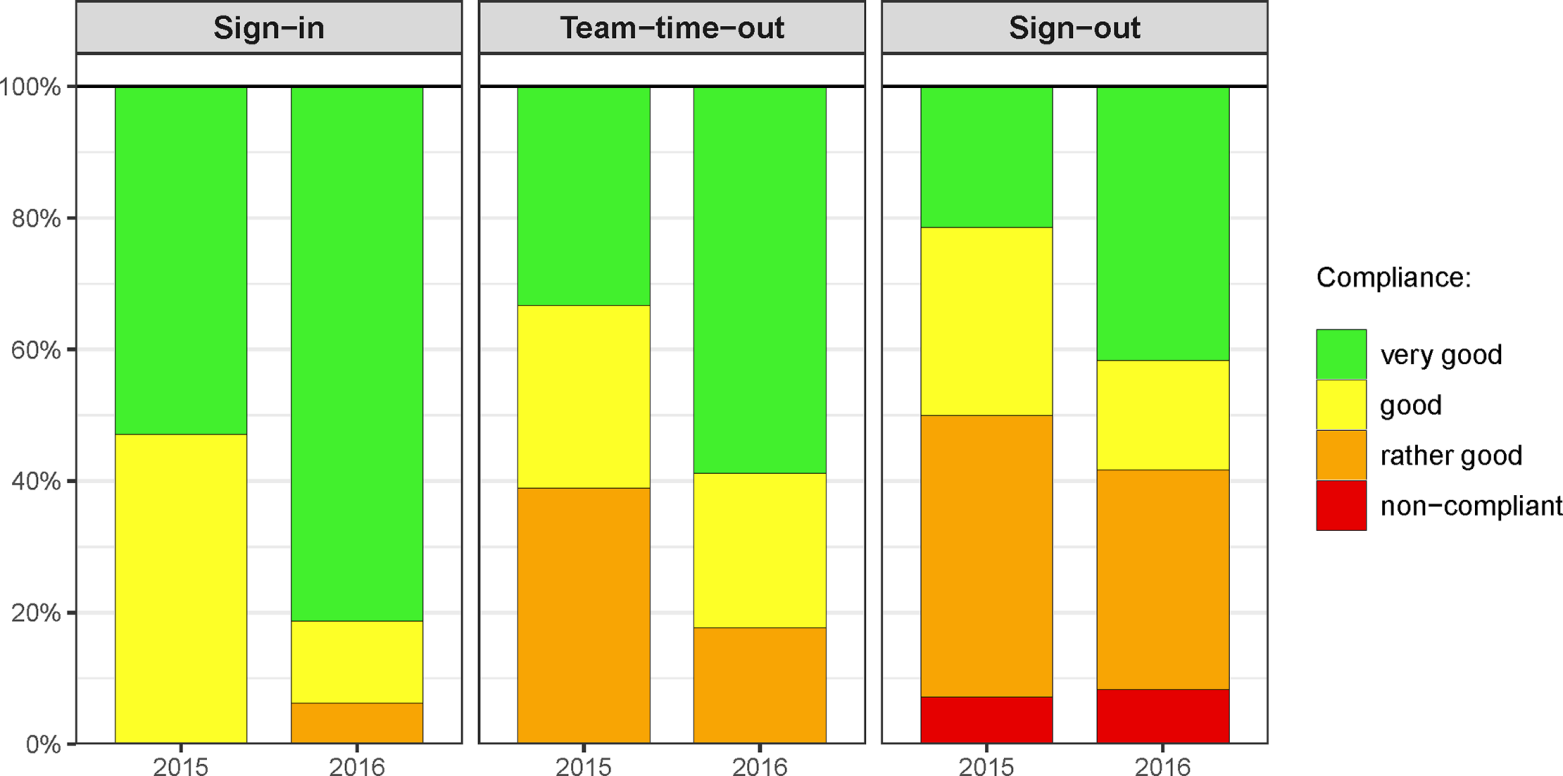
Results of snapshot audits.

The observation day for snapshot audits was announced a week in advance to the directors of involved departments/divisions. In 2015, snapshot audits were performed between May and October. In 2016 snapshot audits were performed between February and August. OR teams were unaware of which of the SSC-procedures were reviewed on that respective day. Each observation team started in the morning, and observed one to five operating theatre sessions per day. Incomplete auditing (e.g. observers switching to another room to log as many entire SSC processes as possible) was also documented (Fig 1).

### Feedback sessions

Immediately after a snapshot audit observers provided oral feedback of their observations to the available OR team members. Each relevant SSC item according to Table 1 and its findings were mentioned and in case of any further questions by the OR team discussed with available OR team members. In general, the OR team consisted of a scrub nurse, anesthesia nurse, anesthesiologist, surgeon(s) and the circulating nurse. However, the number of OR team members who were present during the oral feedback varied, eg. when an oral feedback was given after the TTO, all OR team members were present. In cases when oral feedback was given after the SO, some of the OR team members already left the OR. The length of oral feedback sessions ranged from 3 to 5 minutes depending on the number of observed SSC application errors and encountered questions. Oral feedback sessions were not recorded, however, application errors were written down by observers using the checklist (Table 1). No individual scoring of each observer was performed alone, only after the SSC phase, scores were marked in joined agreement of the findings. These documented results of snapshot audits were then transformed into an electronic database (EvaSys Version 6.0, Healthcare Survey Automation Suite, Electric Paper Evaluationssysteme GmbH, Lüneburg, Germany). Automated reports were then generated and forwarded to the respective nursing and surgical directors of the involved operative divisions/departments as well as to the corresponding divisional directors of the department of anesthesiology. The report included the three checkpoint items alongside with the rating on the 4-point-Likert-scale of each observed day as well as all application errors in a narrative manner which were observed during the snapshot audits. By providing the report to the respective nursing and surgical directors we encouraged to comment on the observers’ findings via email in free written form via the reply function of the hospital mail management system (no additional evaluation sheet or scoring).

### Statistical analysis

The audit day ratings were summarized as absolute and relative frequencies in each year. Missing ratings for some of the phases on a particular day were ignored. For inductive analyses, available audit day ratings were dichotomized into “very good compliance” versus the other three categories. The proportion of SSC phases rated as having “very good compliance” was then compared between the two years with Fisher’s exact test. All statistical analyses were performed using R version 3.3.3.

## Results

136 surgical interventions (2015: n = 67; 2016: n = 69) were audited on a total of 36 days: 19 and 17 days in 2015 and 2016, respectively. For all observed surgical interventions within a department, a report was compiled which included all observers’ findings for the respective snapshot audit day. The results of the snapshot audits are shown in Fig 1.

### Sign-in

The Sign-in showed a “very good compliance” in 52.9% (9/17) of observed cases in 2015, which increased to 81.25% (13/16) in 2016. This difference was not statistically significant (p = 0.141). Ratings were available for 89.5% (17/19) of audit days for the SI in 2015 and for 94.1% (16/17) of days in 2016, respectively.

### Team-time-out

The Team-time-out showed a “very good compliance” in 33.3% (6/18) of observed cases in 2015, and increased to 58.8% (10/17) in 2016. Again, this difference was not statistically significant (p = 0.181). Ratings were available for 94.7% (18/19) of audit days for the TTO in 2015 and for 100% of days in 2016, respectively.

### Sign-out

The Sign-out showed a “very good compliance” in 21.4% (3/14) of observed cases in 2015, and again this number increased in 2016 to 41.7% (5/12). However, this difference, too, was not statistically significant (p = 0.401). Ratings were available for 73.7% (14/19) of audit days for the SO in 2015 and for 70.6% (12/17) of days in 2016, respectively.

### Qualitative snapshot audit findings

Overall reasons for inconsistencies when applying the SSC are shown in Table 2. During the three phases of the SSC, it was observed that certain checklist items were not properly checked or, if checked, they were not ticked-off in the SSC. Most often, the wrong time-window for performing the SSC was chosen.

**Table 2:** Errors observed during snapshot audits. Numbers in brackets are percentages of all observer remarks in that year.

Observed errors in using the SSC	2015 (N = 54)	2016 (N = 25)
**Sign-in**		
Site was not marked		1
Equipment was checked but checklist item was not ticked off	1	
Equipment was not tested		3
SI was performed but not documented	4	4
**SI-error rate**	**5 (9.3%)**	**8 (32.0%)**
**Team-time-out**		
TTO was performed but checklist items were not ticked off		1
TTO was done too early (not all team members were in the OR)	16	6
Team members did not focus on TTO	2	1
Checklist items of the SI were partially ticked off during TTO		1
TTO was not stopped despite mentioned operative site mismatch	1	
Checklist items of TTO already ticked off during SI	1	
Team did not check all checklist items	8	1
No answer from the team but checklist items were ticked off	2	
SSC coordinator asked and answered the checklist item	2	
**TTO-error rate**	**32 (59.3%)**	**10 (40.0%)**
**Sign-out**		
SO was performed during skin closure	4	1
SO was performed after skin closure	4	5
Team did not check all checklist items	4	
A team member leaves the OR before the SO	4	
No SO	1	1
**SO-error rate**	**17 (31.5%)**	**7 (28.0%)**

Individual oral feedback which was given immediately after the snapshot audit of an observed surgical intervention by observers to the available OR team members was brief and in all cases no further feedback or concerns were provided by the OR team members to observers. Additionally, the written report was sent to the heads/directors of the involved departments/divisions via email (n = 19; n = 17), and two email replies were noted in 2015 (response rate: 11%) and three email replies in 2016 (response rate: 16%).

## Discussion

A major factor of the SSC use is its correct and consistent implementation. It was shown that increased SSC compliance correlates with reduced complication rates and improved patient outcomes [20]. To detect SSC compliance several methods have become available, one is, for example, the unannounced retrospective quantitative evaluation of paper-based forms [10, 11]. While quantitative evaluation of forms aids in assessing documentation habits, it cannot provide insights into the actual application in realtime. This is where qualitative assessments come into place, and direct observations may well be a warranted means in doing so [18]. The herein presented qualitative review of the SSC by using snapshot audits demonstrated improvements over the observation period during all three phases of the SSC, however, these improvements were not statistically significant. The qualitative review also revealed application errors when applying the SSC, of which the missing documentation of an actually performed item (SI) or the wrong timing for an item (TTO, SO) were most common. These observations were readily missed in previous assessments of the SSC (1,2).

Oral feedback immediately after the snapshot audit provided the opportunity to OR team members to reflect on their habitual process of using the SSC as assessed by independent observers. While independent members of a different organisational unit can provide an unbiased approach during observations, this can also lead to misunderstandings among the observers and the observed: for example, as a direct result of the different organizational functions (managerial executive department vs. medical/allied-health professional level). Furthermore, the observers in our study have had previous experience as operating theatre personnel, but the provisional training for the study observations did not comprise feedback or communication techniques. Additional written feedback was provided to directors of involved departments/divisions to encourage active debate of the SSC’s use among the professional OR groups without external involvement and opportunity to provide further feedback to the executive department, which performed the snapshot audits. Our results detected positive trends indicating improvement of the SSC’s use (for all three phases) from 2015 to 2016, and we hypothesized that the (direct) oral feedback and written reports to directors (to encourage inner team debate) were part of the success story. However, the received written qualitative feedback of involved departments/divisions failed to support this theory as only a small proportion responded to the written report.

Russ et al. also revealed large variation when using the WHO-SSC. In 97.5% a TTO was performed, however, in just 33.3% of all cases a SO was done and the authors also stated that the recommended guidelines for its use were not followed in the majority of cases [21]. According to our findings we also observed this type of variation during the SI, TTO and SO.

Since 2011 we encourage the correct use of the SSC [10]. Furthermore, in an in-house survey in 2015, 99.4% healthcare professionals stated that they used the SSC. Also, the estimation of individual perception of the SSC's usefulness showed that the use of the SSC was rated as rather easy, familiar, generally important, and good for patients as well as for employees. Only comfort of use was rated low [11]. An unannounced audit, where SSCs were collected to check if all SSC-items were ticked off, showed that SSCs were used in 93.1% of operations, and the completion rate, corresponding to ticked off checklist items, was 57.2% [11]. However, we assumed that not ticking off certain checklist items does not necessarily imply that a team was not performing the SSC [11]. Previous snapshot audits confirmed that not ticked-off checklist items were generally associated with its poor application [20]. Interestingly, our qualitative results revealed that actually performed checklist items were not properly ticked, demonstrating a contrast between performed tasks and their documentation. This is also in support of the methodology of snapshot audits to evaluate the SSC application, which aided in revealing sources of application errors as well as non-compliance. Interestingly, the SI was performed very well and improved during the observation period as compared to the following SSC-phases. One possible explanation for this phenomenon is that the SI concerns only one professional group and does not require interaction with other professional groups in the OR, which the TTO and SO do. This was confirmed by our observation of the TTO, which was mostly performed at the right time, but not all team members focussed on the SSC, the background was noisy, or checklist items were not properly checked. The SO’s performance was the poorest in our observation. In general, whether the poor teamwork and ineffective communication of OR teams had any implications in adverse events in patients was not part of this work [22–25].

Described as the Hawthorne effect, observational results can be biased: teams tend to follow procedures more rigorously when they know they are being observed [21, 26, 27]. However, our results demonstrated that observing healthcare professionals during snapshot audits apparently did not influence their habits when performing the SSC as shown by frequently observed errors.

What can be done to improve the compliance rate when using the SSC? Snapshot audits gave valuable insight and highlighted possibilities to increase SSC compliance and acceptance among healthcare professionals. Our results revealed positive trends in the SSC’s application during snapshot audits, and a lack of (qualitative) feedback from OR team members or its directors. We suppose that the lack of options for bilateral communication was one hindering factor for constructive feedback from/among OR teams or its directors. This can be addressed in the future by providing additional training to observers performing the snapshot audits and offering structured feedback options to the OR teams (eg. anonymous paper-based/ web-based forms, short structured feedback interviews performed by observers). Another option to address SSC application errors and raise awareness of its application is to customize the training approach to staff applying the SSC in the OR. The customization has been proven to be more efficient than the mere distribution of WHO’s standard checklists [28, 29]. A further approach is to integrate the SSC into the teams’ workflow instead of providing another add-on hindering the routine processes in the operating theatre. As an example, the scrapping of paper-based forms and integration into the electronic health-care records of the hospital system, even by the use of vocal commands, demonstrated additional benefits: ubiquituous attention, no additional paper forms, integration in decision support system (eg. automatically retrieving patient demographics, updated laboratory values, allergies, medications and further specimens and it audibly verbalized the checklist) [30, 31]. This can be realized by proper cooperation of clinical teams with an efficient IT support [31]. Last but not least, snapshot audits proved to be a valuable tool to detect not only application errors of the SSC, but also observation errors readily missed by performing retrospective checklist reviews alone.

A limitation of this study was the lack of a priori sample size calculation, resulting from the pilot character of snapshot audit implementation with incorporated feedback sessions in our university hospital. Furthermore, observers were unable to observe each relevant step for each patient of each of the selected days. Observers switched from one OR to the other after e.g. a TTO was performed, in order to use the time for further observations instead of waiting for the SO. Moreover, observers provided observational reports only per observed day, and the oral feedback to/from OR team members was not performed in a structured way to allow for quantifiable evaluation. A further limitation was the gap between the first and second snapshot audit as the feedback after the first snapshot audit might have had only a short-term impact. Additionally, the lack of engagement by clinicians and executives in the feedback suggests that as an intervention the feedback was not very likely to have a long-term impact on practice. Finally, since more than 900 healthcare professionals work within the observed ORs, snapshot audits only involved a portion of these. Therefore, we cannot rule out any secular trends or team member composition being responsible for any observed changes between 2015 and 2016.

## Conclusions

In conclusion, snapshot audits made a qualitative analysis of actual SSC practices possible, and revealed that its compliance was not as bad as implied by the mere check of completeness of ticks in paper-based documents. Repeated snapshot audits helped to identify areas for improvement of using the SSC and to better understand the challenges, when evaluating SSC application and its compliance. Our study results suggest that bilateral feedback may encourage further discussion of the SSC’s application and its evaluation, but requires structured and quantifiable feedback options. This should be integrated in the snapshot methodology workflow by provision of feedback training to observers and via standardized feedback forms for OR staff or its directors (eg. paper-based or online). Further areas for improving the SSC application include the customization of its training for OR staff, and IT supported integration of the SSC in the electronic patient record system (eg. providing integration in routine processes and even creating decision-support systems based on the SSC). In our point of view, snapshot audits are a very useful tool to monitor the introduction of new methods in regularly performed workflows to improve understanding of barriers and facilitators from a quantitative and qualitative perspective.
